# A physiotherapist-delivered integrated exercise and pain coping skills training intervention for individuals with knee osteoarthritis: a randomised controlled trial protocol

**DOI:** 10.1186/1471-2474-13-129

**Published:** 2012-07-24

**Authors:** Kim L Bennell, Yasmin Ahamed, Christina Bryant, Gwendolen Jull, Michael A Hunt, Justin Kenardy, Andrew Forbes, Anthony Harris, Michael Nicholas, Ben Metcalf, Thorlene Egerton, Francis J Keefe

**Affiliations:** 1 Centre for Health, Exercise & Sports Medicine, Department of Physiotherapy, University of Melbourne, Melbourne, VIC, Australia; 2 Psychological Sciences, University of Melbourne, Melbourne, VIC, Australia; 3 Centre of Clinical Research Excellence in Spinal Pain, Injury & Health, School of Health and Rehabilitation Sciences, University of Queensland, Brisbane, Qld, Australia; 4 Department of Physical Therapy, University of British Columbia, Vancouver, BC, Canada; 5 Centre of National Research on Disability and Rehabilitation Medicine, School of Psychology and Medicine, University of Queensland, Brisbane, Qld, Australia; 6 Department of Epidemiology and Preventative Medicine, School of Public Health and Preventative Medicine, Monash University, Melbourne, VIC, Australia; 7 Centre for Health Economics, Monash University, Melbourne, VIC, Australia; 8 Pain Management and Research Centre, University of Sydney, Sydney, NSW, Australia; 9 Department of Psychiatry and Behavioral Sciences, Duke University School of Medicine, Durham, NC, USA

## Abstract

**Background:**

Knee osteoarthritis (OA) is a prevalent chronic musculoskeletal condition with no cure. Pain is the primary symptom and results from a complex interaction between structural changes, physical impairments and psychological factors. Much evidence supports the use of strengthening exercises to improve pain and physical function in this patient population. There is also a growing body of research examining the effects of psychologist-delivered pain coping skills training (PCST) particularly in other chronic pain conditions. Though typically provided separately, there are symptom, resource and personnel advantages of exercise and PCST being delivered together by a single healthcare professional. Physiotherapists are a logical choice to be trained to deliver a PCST intervention as they already have expertise in administering exercise for knee OA and are cognisant of the need for a biopsychosocial approach to management. No studies to date have examined the effects of an integrated exercise and PCST program delivered solely by *physiotherapists* in this population. The primary aim of this multisite randomised controlled trial is to investigate whether an integrated 12-week PCST and exercise treatment program delivered by physiotherapists is more efficacious than either program alone in treating pain and physical function in individuals with knee OA.

**Methods/design:**

This will be an assessor-blinded, 3-arm randomised controlled trial of a 12-week intervention involving 10 physiotherapy visits together with home practice. Participants with symptomatic and radiographic knee OA will be recruited from the community in two cities in Australia and randomized into one of three groups: exercise alone, PCST alone, or integrated PCST and exercise. Randomisation will be stratified by city (Melbourne or Brisbane) and gender. Primary outcomes are overall average pain in the past week measured by a Visual Analogue Scale and physical function measured by the Western Ontario and McMaster Universities Osteoarthritis Index subscale. Secondary outcomes include global rating of change, muscle strength, functional performance, physical activity levels, health related quality of life and psychological factors. Measurements will be taken at baseline and immediately following the intervention (12 weeks) as well as at 32 weeks and 52 weeks to examine maintenance of any intervention effects. Specific assessment of adherence to the treatment program will also be made at weeks 22 and 42. Relative cost-effectiveness will be determined from health service usage and outcome data.

**Discussion:**

The findings from this randomised controlled trial will provide evidence for the efficacy of an integrated PCST and exercise program delivered by physiotherapists in the management of painful and functionally limiting knee OA compared to either program alone.

**Trial registration:**

Australian New Zealand Clinical Trials Registry reference number: ACTRN12610000533099

## Background

Knee osteoarthritis (OA) is a prevalent chronic musculoskeletal condition [1] associated with pain, physical and psychological dysfunction, and reduced quality of life in affected individuals [2,3]. In addition to the personal burden of knee OA, there are substantial direct and indirect health care costs making knee OA a major public health problem [4]. Given the extent of the problem and the fact that the prevalence of OA will escalate with the ageing population and increases in obesity rates [5], effective treatment strategies are required. In particular strategies that promote long-term self-management are important given the chronicity of the condition.

Pain is the primary symptom of knee OA and people with higher levels of pain have lower levels of physical function, greater functional decline and reduced quality of life [6]. Individuals with painful knee OA experience difficulty performing basic daily activities such as shopping, performing household chores, stair climbing as well as engaging in social and outdoor activities [7]. Furthermore, knee pain related to OA is one of the strongest predictors of employment status and productivity [8]. Reducing pain is therefore a relevant and important treatment aim for this patient group.

The experience of pain is influenced by a multitude of structural, physical, and psychosocial factors [6,9,10]. Whilst stimulation of nociceptors in the capsule, subchondral bone, ligaments and other joint tissues contribute to the perception of pain, structural damage in knee OA is in fact not well correlated with pain severity [11]. Instead, physical and psychological impairments that are commonly found in this patient population are more important predictors of pain. Muscle weakness, particularly of the quadriceps, is associated with higher levels of pain and greater declines in physical dysfunction [10,12]. Psychological impairments including pain catastrophising [13], poor pain coping strategies [14], anxiety [15], depression or depressed mood [15,16] and social isolation [16] are also related to increased pain levels in people with knee OA. Furthermore, bi-directional relationships exist between pain and physical and psychological impairments whereby pain can influence, and in turn be influenced by these factors [17], leading to a downward cascade in physical and mental functioning. Given the importance of both physical and psychological impairments, it would seem logical that treatments should address both aspects in order to maximise patient outcomes.

Clinical guidelines advocate conservative non-drug strategies for the treatment of knee OA [18,19]. In particular, exercise including muscle strengthening is recommended [20] and is supported by considerable research evidence [21,22]. A recent systematic review included 18 randomised controlled trials, the majority of which involved home-based programs of quadriceps or lower limb muscle strengthening. The results showed significant improvements in pain and self-reported physical function with muscle strengthening exercise [22]. However, despite consistent findings of short-term improvements with exercise, reduced adherence to exercise programs limits long-term effectiveness [21,23]. Thus, interventions that facilitate long-term exercise adherence are needed.

Whether strengthening exercise also improves psychological parameters in people with knee OA is less clear as few studies include adequate assessment of such parameters [24-26]. There is some evidence from randomised controlled trials that strengthening exercise is associated with reductions in depressive symptoms [27] in those with knee OA [28] and in other chronic conditions [29,30]. Such exercise has also been shown to improve self-efficacy, fatigue symptoms, and sleep quality in depressed older adults [31]. Based on this limited evidence, it appears that strengthening exercise may improve some aspects of psychological functioning in those with knee OA but further research is needed.

As psychological factors are related to pain, psychological interventions are worthy of attention. Of the psychological interventions that have been considered, pain management, based on the principles of cognitive behavioural therapy, is the most extensively researched intervention in chronic pain conditions and has the strongest evidence base. One such approach, Pain Coping Skills Training (PCST) focuses on self-management and is well recognized as an effective cognitive behavioural treatment for disease-related pain conditions [29,30,32,33]. However, a meta-analysis identified only two clinical trials involving PCST in those with knee OA. Both trials showed improvements in pain and physical function over a 12 week intervention period [24,34,35] with benefits appearing to diminish over time. Given the potential benefits of PCST and yet the limited evidence in people with knee OA, further research is needed to investigate its efficacy.

In accordance with a biopsychosocial approach to the management of chronic pain [36] both physical and psychological impairments should be addressed in people with knee OA. The studies that have tested integrated exercise and psychological treatments in a variety of other chronic conditions including cancer [32,33], low back pain [29] and fibromyalgia [30] have shown positive results. Only one study has examined the effects of a combined program of PCST and exercise on pain in those with knee OA [24]. This study compared a 12 week intervention of spouse-assisted PCST alone, exercise training alone, spouse-assisted PCST and exercise training or standard care in 72 participants with knee OA [24]. The results showed that the combined program improved physical fitness, strength, pain coping and self-efficacy. In addition, those with improvements in self-efficacy were more likely to improve in psychological functioning. However, the combined intervention required participants to attend a total of four hours per week of therapy delivered by two different health professionals, in addition to home practice. From a practical perspective this requires a considerable time commitment from patients and involves substantial treatment costs that would not necessarily be sustainable in everyday practice. Research investigating other methods of delivering combined treatments is required.

PCST is generally delivered by a psychologist specialising in pain management. However, it may be beneficial to utilise a single health care professional such as a physiotherapist to deliver an integrated physical and psychological intervention [37,38]. Potential advantages of using a single therapist include better integration of the intervention components, increased availability of PCST treatment to those who may not have access to a psychologist, reduced time and cost for patients and reduced overall costs to the health care system. Although physiotherapists do not have formal training in pain management they are a logical choice to be trained in the delivery of PCST given their expertise in administering physical treatments to treat pain and their understanding of the biopsychosocial approach. No studies to date have examined the effects of an integrated exercise and formal PCST program delivered by physiotherapists in this patient population.

This project primarily aims to compare the efficacy of a 12-week integrated PCST and exercise program delivered by physiotherapists to treat pain and physical function in individuals with knee OA compared to PCST or exercise programs alone. The secondary aims of the study are to compare the efficacy of these programs on functional performance, psychological parameters, quality of life, muscle strength, physical activity levels and cost-effectiveness and to examine longer-term outcomes over a 9-month follow up period.

### Primary hypothesis

H1: A 12-week integrated intervention of exercise and PCST will be more efficacious in improving pain and self-reported physical function than a 12-week intervention of either PCST or exercise alone immediately following the intervention.

### Secondary hypotheses

H2: An integrated intervention of exercise and PCST will be more efficacious in improving pain and self-reported physical function than either PCST or exercise alone at 32 weeks and 52 weeks.

H3: An integrated intervention will be more efficacious in improving psychological function, functional performance, quality of life, physical activity levels and perceived response to treatment than either PCST or exercise alone immediately following the intervention and at 32 weeks and 52 weeks.

H4: Exercise will lead to greater improvements in muscle strength than PCST; PCST will lead to greater improvements in psychological parameters than exercise; and an integrated intervention will lead to greater improvements in both strength and psychological parameters at measured time points.

H5: Adherence to exercise during the 9-month unsupervised follow-up period will be greater with an integrated intervention than with an intervention of exercise alone.

H6: An integrated intervention will be more cost-effective than an intervention of exercise or PCST alone when costs are compared and related to the effects of the intervention at 52 weeks.

H7: Specific patient baseline characteristics will moderate or predict treatment effects while pre- to post-treatment changes in the targeted cognitions, behaviours and physical impairments will mediate the effects of PCST and exercise on subsequent patient pain and disability.

## Methods/design

### Trial design

This will be an assessor-blinded, 3-arm randomised controlled trial of a 12-week intervention involving 10 physiotherapy visits together with home practice. Measurements will be taken at baseline and immediately following the intervention (12 weeks) as well as at 32 weeks and 52 weeks to examine maintenance of any intervention effects. Specific assessment of adherence to the treatment program will also be made at weeks 22 and 42. The study will be conducted at two sites, Melbourne and Brisbane, Australia to facilitate generalizability of the results and to ensure timely recruitment. The protocol will conform to CONSORT guidelines for reporting non-pharmacological interventions [39] and has been registered with the Australia and New Zealand Clinical Trials Registry prior to study commencement.

### Participants

We will recruit participants with painful knee OA from the community in the Melbourne and Brisbane metropolitan regions. A number of recruitment strategies will be used including (i) advertising through local clubs, community centers, newspapers, Australian Health Management, Arthritis Australia and University websites, University staff newsletters, radio, and Facebook; (ii) placing brochures and study posters in medical and physiotherapy clinics; (iii) conducting presentations about knee OA in the local community, and (iv) using our database of people who have been recruited from the community for prior studies and have given consent for future contact.

To be eligible, participants must fulfill the following criteria:

i. Aged ≥ 50 years;

ii. Knee OA fulfilling American College of Rheumatology classification criteria [40] of knee pain on most days of the past month and tibiofemoral osteophytes on x-ray (Kellgren and Lawrence ≥ Grade 2) [41];

iii. Knee pain for ≥ 3 months;

iv. Overall average knee pain in the last week ≥ 40 on a 100 mm Visual Analogue Scale (VAS);

v. Western Ontario and McMaster Universities (WOMAC) Osteoarthritis Index physical function score of ≥ 25 indicating at least a moderate level of difficulty in performing activities of daily living.

The exclusion criteria are:

i. Knee surgery including arthroscopy within the past 6 months;

ii. Awaiting or planning any back or lower limb surgery within the next 12 months;

iii. Current or past (within 3 months) oral or intra-articular corticosteroid use;

iv. Systemic arthritic conditions such as rheumatoid arthritis;

v. Physiotherapy, chiropractic or acupuncture treatment or exercises specifically for the knee within the past 6 months;

vi. Walking >30 min continuously daily or participating in a regular (more than twice a week) exercise program;

vii. Past participation in a PCST program;

viii. Inability to walk unaided as this is necessary for some of the physical testing;

ix. Medical condition precluding safe exercise such as uncontrolled hypertension or heart condition;

x. Self-reported psychiatric history such as schizophrenia;

xi. Self-reported diagnosis of current clinical depression;

xii. Neurological condition such as Parkinson’s disease, Multiple sclerosis or stroke;

xiii. Inadequate written and spoken English;

xiv. Unable to comply with the protocol such as inability to attend therapy sessions or attend assessment appointments at the University.

### Procedure

**Figure 1:**
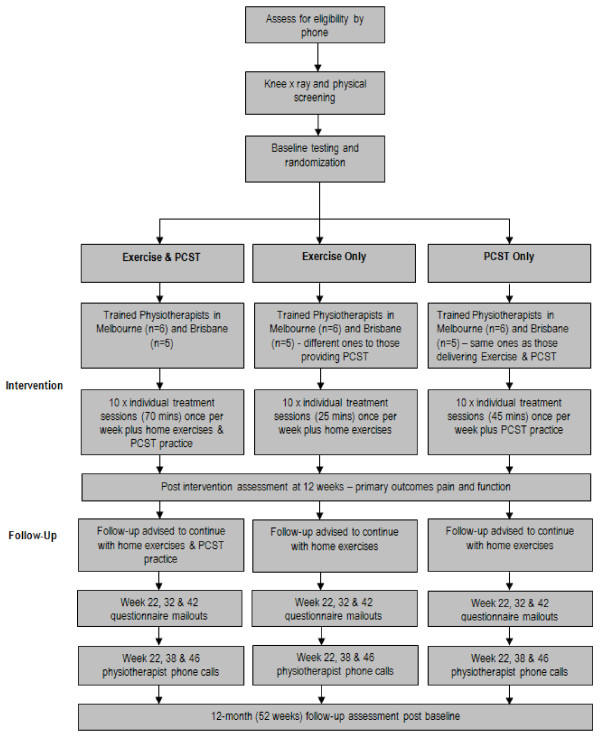
**Diagram of study protocol** .

The procedure is outlined in Figure 1. Preliminary screening will be conducted over the telephone by a research assistant not involved in outcome assessment. Volunteers who are deemed potentially eligible will undergo a semiflexed posteroanterior x-ray of their painful knee (the more symptomatic knee in cases of bilateral eligible knee pain) at one of six trial radiology centres unless they can provide their own such films from within the previous 12 months. X-ray grading will be performed by two trained researchers at each site and any disagreement will be resolved through discussion or where necessary, by a third rater. A screening record will be maintained to document the criteria eliminating those found to be ineligible. Participants will attend the University of Melbourne or the University of Queensland for baseline testing, following which they will be randomised into one of three intervention groups: (i) exercise; (ii) PCST; or (iii) integrated exercise and PCST. Each intervention will last for 12 weeks and will involve 10 individual visits to a project physiotherapist together with home exercise and/or pain coping skills practice. Following the intervention, participants will continue their home exercise and/or pain coping skills practice unsupervised for nine months. Participants will be re-assessed at week 12 (immediately following the intervention), at week 32 (by postal questionnaires) and at week 52 (at the University). Additional questionnaires relating to home practice adherence will also be collected at weeks 22 and 42. Participants will also wear a pedometer for 7 consecutive days at baseline, 12 weeks and 52 weeks. All participants will be asked to refrain from seeking other forms of treatment during the trial. However, due to ethical considerations, analgesia and non-steroidal anti-inflammatory drugs will be permitted as required.

Ethics approval has been obtained from the University of Melbourne Human Research Ethics Committee (HREC: 1033341) and Radiation Safety Human Services and from the University of Queensland Medical Research Ethics Committee (MREC: 2010000340) and Radiation Protection Advisor. All participants will provide written informed consent.

### Blinding

The outcome assessors at both sites will be blind to group allocation and will not be involved in providing the interventions nor will they visit any of the physiotherapy treatment centers. Participants will be requested not to disclose details about their treatment to the outcome assessors. The physiotherapists as well as the psychologists and researchers managing the study at both sites are by necessity unblinded. The statistician will be blind to group allocation until completion of the statistical analyses.

### Randomisation and allocation concealment

The randomisation schedule will be prepared by the study biostatistician using a computer generated random numbers table. Randomisation will be conducted by random permuted blocks of size from 4 to 6 stratified by site (Melbourne or Brisbane) and gender (male or female). To conceal randomisation, an independent staff member will prepare consecutively numbered, sealed, opaque envelopes for each site. The envelopes will be kept in a locked location at each site accessible only by an unblinded researcher. Each envelope will be opened in sequence once the participant has completed the baseline measurements by a person not involved in participant recruitment. An unblinded researcher will then schedule the participants’ first appointment with their treating physiotherapist.

### Physiotherapists

Twenty-one experienced physiotherapists, 11 in Melbourne (six delivering both PCST only and integrated interventions, five delivering the exercise only intervention) and 10 in Brisbane (five delivering both PCST only and integrated interventions, five delivering the exercise only intervention), with at least five years of musculoskeletal clinical experience and located at various metropolitan private practices will be trained to deliver the interventions. Two of the PCST physiotherapists in Melbourne have prior experience with the delivery of PCST interventions and regularly use a similar form of this treatment in their practices. To ensure no carry over effects from training in PCST, the physiotherapists delivering the exercise only intervention will not be trained to provide the PCST. This large number of treating physiotherapists is necessary for practical reasons and to improve the generalisability of the results.

### Psychologists

Four clinical psychologists (two at each of the Melbourne and Brisbane sites) will be responsible for the ongoing PCST training and monitoring of the physiotherapists who are involved in the delivery of PCST. These include one senior psychology researcher at each site who will oversee and guide one site psychologist. The two site psychologists will be responsible for the ongoing training and monitoring of the physiotherapists. Both have more than five years of clinical experience and specialise in pain management.

### Training of physiotherapists

The physiotherapist training to deliver the PCST intervention will involve an initial 4-day workshop facilitated by a psychologist and pain management specialist (FK) who developed the PCST program. The physiotherapists will then participate in regular tutorials and role-playing with the site psychologists as well as individual practice with an independent person acting as a patient. The physiotherapists will be accredited to deliver the 10 PCST treatment sessions once audio-recordings of each practice session are reviewed by the site psychologist and meet pre-specified criteria for content and quality of delivery.

All 21 physiotherapists will be trained to deliver the exercise program. Training will comprise a 4-hour practical workshop conducted by musculoskeletal physiotherapists. The physiotherapists will be provided with a detailed study manual and a DVD of the training workshop.

### Interventions

Participants in each intervention group will attend 10 individual treatment sessions with a project physiotherapist. The timing of the sessions is approximately once per week. This reflects a realistic dosage in clinical practice and is indicative of the timeframe needed for exercise and PCST effects. Participants will choose the physiotherapist to attend based on the geographical availability of therapists trained to deliver their assigned intervention. Treatment will commence within one week of the baseline assessment.

#### *Exercise intervention*

This will be a home-based exercise program designed to strengthen lower limb muscles and incorporates exercises commonly used in clinical practice. It is based on clinical trials showing that such exercise programs improve pain and function [42]. Six exercises aimed to strengthen the quadriceps, hamstrings, and hip abductor muscles will be taught to participants by the physiotherapist (Table 1). At each of the treatment sessions, the physiotherapist will monitor proper performance and exercise intensity and progress the exercises, if necessary. Intensity will be determined by the participant’s ability to complete 10 repetitions for a given exercise and by perceived difficulty (using a modified Borg scale for resistance training [43]). The participant will be asked to perform the prescribed home exercises four times weekly aiming for a dosage of 3 sets of 10 repetitions. Weights and resistance elastic bands as well as handouts describing the exercises will be provided. Each physiotherapy treatment session will be 25 mins in length (Figure 1, Table 2).

**Table 1:** Description of the exercise program

**Exercise**	**Description and progression**
Knee extensor strengthening	Seated knee extensions with ankle weights. Ankle weights progressed.
Hip abductor strengthening	Level 1: Side lying with ankle weights. Ankle weights progressed.
	Level 2: Standing hip abduction with elastic band around ankles. Elastic band resistance progressed.
Weight-bearing knee/hip extensor strengthening	Level 1: Partial wall squats (option to add elastic band around knees to incorporate hip abductor muscles).
	Level 2: Sit-to-stand (option to add elastic band around knees to incorporate hip abductor muscles).
	Level 3: Split sit-to-stand (or split partial wall squats) – most weight bearing on affected side.
Knee flexor strengthening	Seated knee flexion against elastic band resistance. Elastic band resistance progressed.
Step Ups/Step Down	Progress by increasing the height of the step then adding weight (i.e., back pack or hand weights).

All exercises must be progressed during the program. Dosage is 3 × 10 repetitions with 5 second holds for all exercises with the exception of level 2 and 3 sit to stand exercises which have 3 second holds.

**Table 2:** Overview of pain coping skills training (PCST) and exercise components

**Pain coping skills training**	**Exercise**
**Session 1**	**Session 1**
· Introduction and discussion of patient assessment form	· Introduction and discussion of patient assessment form
· Patient education about knee OA and treatment	· Patient education about knee OA and treatment
· Teach patient weekly home PCST practice	· Teach patient home exercises
· Home practice prescribed daily	· Home exercises prescribed 4 times/week
· Home practice prescribed daily	· Discuss patient log book and attendance
**Session 2-10**	**Session 2-10**
· Review of previous week	· Review of previous week
· Teach patient new pain coping skill	· Check and progress patient home exercises
· Check and progress patient home practice	· Home exercises prescribed 4 times/week
· Home practice prescribed daily	· Check patient log book
· Check patient log book and set goals for the week	
**Follow-up period**	**Follow-up period**
· Home practice prescribed as required	· Home exercises prescribed 3 times/week

#### *Pain Coping Skills Training (PCST) intervention*

The PCST program has been designed to specifically enhance the participants’ ability to employ behavioral and cognitive pain coping strategies aimed at increasing self-efficacy and decreasing pain and pain catastrophising. The program involves 10 weekly modules (Table 3) and is similar to those used in previous studies in knee OA [24,34]. Interactive sessions will emphasise the participants’ understanding of the neuromechanical processes of pain which underscores the role that PCST can play as well as provide training in a number of pain coping skills (activity-rest cycling, pleasant activity scheduling, problem solving, identifying negative thoughts, challenging negative thoughts, developing coping thoughts, pleasant imagery, counting backwards, and auditory stimulation) and in practical ways of applying newly developed coping skills. The participant will be asked to perform the prescribed home PCST practice daily with the dosage dependent on the actual skill taught during the particular week. Each physiotherapy treatment session will be 45 mins in length (Figure 1, Table 2).

**Table 3:** Description of the Pain Coping Skills Training (PCST) Intervention

**PCST session**	**Content**	**Home practice dosage**
Session 1: Progressive Muscle Relaxation (PMR)	- Introduce gait control theory	2 PMR practices per day
	- Provide rationale for pain coping skills training	
	- Train participant in PMR	
Session 2: Mini-Practices	- Review PMR	10 or more mini-practices per day
	-Train participants on mini-practices	
Session 3: Activity-Rest Cycling	- Review PMR and mini-practices	Use technique twice per week
	- Introduce activity-rest cycling	
Session 4: Pleasant Activity Scheduling	- Describe how pleasant activity scheduling can be used to control and decrease pain	3 pleasant activities per week
	- Set pleasant activity goals with participant	
	- Discuss how to use mini-practices and activity-rest cycling in achieving pleasant activity goals.	
Session 5: Identifying Negative Thoughts, Thought Records	- Present cognitive model (ABC Model-how an event leads to automatic thoughts and result in certain consequences)	Record situations and thoughts daily
	- Teach participant how to use thought records to monitor negative thoughts	
Session 6: Challenging Negative Thoughts, Calming Self-Statements	- Work with participant to challenge negative thoughts	Practice developing alternative coping thoughts daily
	- Develop calming self-statements	
Session 7: Problem Solving I, Pleasant Imagery and Distraction Techniques I	- Training in problem solving	Problem solving activity: 1 per day
	- Training in pleasant imagery	
	- Training in counting backwards	Pleasant imagery: 2 per day
Session 8: Distraction Techniques II, Review of Skills	- Train use of focal points and auditory stimulation as distraction methods	3 distraction techniques per week
	- Review skills from previous weeks	
Session 9: Problem Solving II (Applying Pain Coping Skills in Problem Situations)	- Identify problem situations	Record situations and thoughts daily
	- Develop coping plans	
Session 10: Coping Skills Maintenance, Early Warning Signs/Developing a Coping Plan	- Review principles of relapse prevention	
	- Identify early warning signs of reduced coping	
	- Develop coping plans to address lapses in coping	

All components of the PCST program are mandatory. Home practice from each session is carried forward into the remainder of the program.

#### *Integrated exercise and PCST intervention*

This intervention will integrate both the exercise and PCST programs described above within a single integrated treatment session. While the therapist will deliver the same PCST program, examples relating to exercise and activity can be used as material for the skills being taught to a greater extent than in the PCST alone group. Participants will be encouraged to incorporate the learned PCST skills into their home exercise program and specific training will be provided on how learned skills can be applied during the exercise performance at the same clinic visit. Each physiotherapy treatment session will be 70 min in length which includes 25 mins of exercise and 45 mins of PCST (Figure 1, Table 2).

### Quality assurance and intervention integrity

Physiotherapist adherence to the PCST protocol and quality of delivery will be monitored and enhanced by regular (approximately fortnightly) team meetings with the site psychologist. Audio-recordings of each physiotherapy treatment session will be reviewed by the site psychologist with feedback provided to the physiotherapist. For the two treatment groups involving PCST, the site psychologists will formally rate approximately 10% of sessions using randomly selected treatment audiotapes. The Melbourne psychologist will rate those from Brisbane and vice versa following a period of training to ensure consistency of ratings. The physiotherapist delivery of the PCST treatments will be rated on two aspects: 1) adherence to the protocol using a yes/no format and calculated to give a percentage score; and 2) quality of the treatment using a 1–5 scale (1 being poor, 2 fair, 3 satisfactory, 4 very good and 5 excellent) for each of the following therapist behaviours: establishes/maintains rapport; remains on schedule with the protocol or makes appropriate adjustments when indicated; applies PCST protocol to participant’s situation and current challenges; encourages participant’s active involvement in the session; uses time effectively/appropriate pacing; demonstrates good interpersonal skills; demonstrates professionalism and clinical judgment; overall effectiveness/skill of the therapist.

In addition, an unblinded research assistant will randomly observe selected treatment sessions for all treatment arms at each physiotherapy clinic and provide feedback to the therapist. Group-specific quality assurance checklists will be completed by the research assistant during the session. The checklist contains items pertaining to the protocol such as therapist time spent solely with the participant, treatment notes completed, review of home practice and handouts provided, through to more complex items such as therapist observation of participant practicing the exercises, therapist use of the rating perceived exertion scale to review intensity of exercises and progression of weights if required and for those in the PCST treatment groups, new concepts introduced and explained. Lastly, participants will complete a questionnaire about their experience with the physiotherapist including whether they would recommend the physiotherapist to someone they knew.

### Follow-up period

During the 9-month unsupervised follow-up period, participants in the exercise only and integrated groups will be requested to continue their home strengthening program but this will be reduced to three times per week. Participants in the PCST and integrated groups will be requested to continue their PCST home practices as needed (Figure 1, Table 2). At weeks 22, 32, 42 and 52 participants will be requested to complete questionnaires posted to them pertaining to adherence to the home program. In addition, the treating physiotherapist will telephone participants at weeks 22, 38 and 46 to discuss progress with the aim of enhancing adherence to the home program. After the 9-month period (week 52), participants will attend a follow up testing session at the University laboratory.

### Measurements

Baseline descriptive data will be obtained by questionnaire and will include age, sex, duration of knee OA symptoms, previous treatment, surgery and medication use for knee OA, employment status, marital status, education level and previous health problems. Radiographic disease severity will be assessed from x-ray using the Kellgren and Lawrence grading system [41]. Measures of height and weight will be taken and body mass index calculated. A summary of all measures collected in the trial are shown in Table 4.

**Table 4:** Summary of measures to be collected

**Primary outcome measures**	**Data collection instrument**	**Collection points**
Average overall pain in past week	100 mm VAS	0, 12, 32, 52 weeks
Self reported physical function in past 48 hours	Physical function subscale WOMAC Osteoarthritis Index 3.1 Likert version	0, 12, 32, 52 weeks
**Secondary outcome measures**		
Pain	Pain subscale WOMAC Osteoarthritis Index 3.1 Likert version	0, 12, 32, 52 weeks
Global rating of change	Overall, for pain and for function - 7 point scale	12, 32, 52 weeks
	Perceived response to treatment - 7 point ordinal scale	12, 32, 52 weeks
Muscle strength	Isometric quadriceps and hamstrings in sitting using a force transducer	0, 12, 52 weeks
	Isometric hip abductors - Hand held dynamometer in supine	0, 12, 52 weeks
Functional performance	Timed 20 m walk	0, 12, 52 weeks
	30 second sit-to-stand	0, 12, 52 weeks
	Timed Up and Go	0, 12, 52 weeks
	Step test	0, 12, 52 weeks
Physical activity levels	Physical Activity Scale for the Elderly (PASE)	0, 12, 32, 52 weeks
	Pedometer worn for 7 days	0, 12, 52 weeks
Health-related quality of life	Assessment of Quality of Life Instrument version 2 (AQoL II)	0, 12, 32, 52 weeks
Self-reported psychological measures	Arthritis Impact Measurement Scale 2	0, 12, 32, 52 weeks
	Arthritis Self-Efficacy Scale	0, 12, 32, 52 weeks
	Arthritis Self-Efficacy for Pain communication Scale	0, 12, 32, 52 weeks
	Pain Self-Efficacy Scale	0, 12, 32, 52 weeks
	Pain Catastrophising Scale	0, 12, 32, 52 weeks
	Coping Strategies questionnaire	0, 12, 32, 52 weeks
	Depression, Anxiety & Stress subscale	0, 12, 32, 52 weeks
	Holding Back Scale	0, 12, 32, 52 weeks
	Patient Health Questionnaire-9	0, 12, 32, 52 weeks
	Self Efficacy for Exercise Scale	0 weeks
	Barriers to Exercise Scale	0 weeks
	Benefits of Exercise Scale	0 weeks
	Barriers and enablers to home exercise	32, 52 weeks
**Other measures**		
Treatment credibility	Treatment Credibility Scale	1, 12 weeks
Treatment session attendance	Therapist treatment records	During intervention
Home practice during treatment	Participant log book – number of days/times completed	Daily during intervention
	Therapist rating of participant adherence using 11-point numeric rating scale	12 weeks
	Self-rated using 11-point numeric rating scale	22, 32, 42, 52 weeks
Home practice during follow up	Questionnaire-number of days performed exercises/pain coping skills in past week	22, 32, 42, 52 weeks
	Questionnaire - usefulness of pain coping skills	32, 52 weeks
Adverse events	Participant logbook	Daily during intervention
	Questionnaire	32, 52 weeks
Healthcare usage and related costs	Questionnaire and health system	0, 12, 32, 52 weeks

#### *Primary outcome measures*

Outcome measures have been selected based on those recommended for clinical trials of OA [44]. The primary outcomes are change in self-reported pain and physical function at 12 weeks.

a) Pain: average knee pain in the past week will be self-assessed using a 100 mm Visual Analogue Scale (VAS) with terminal descriptors of “no pain” and “worst pain possible”. The VAS pain measurement has demonstrated reliability in OA [44].

b) Physical function: this will be self-assessed using the Western Ontario and McMaster Universities Osteoarthritis Index (WOMAC) Likert version 3.1. The physical function subscale has 17 items with a five point Likert response (0 indicating no physical dysfunction, 5 indicating severe physical dysfunction) giving a total score out of 68. The WOMAC is a disease-specific instrument whose validity, reliability, and responsiveness have been demonstrated in an extensive range of OA studies [45].

#### *Secondary outcome measures*

##### Other pain measures

Pain will also be self-assessed using the pain subscale of the Western Ontario and McMaster Universities Osteoarthritis Index (WOMAC) Likert version 3.1. The pain subscale has 5 items with a five point Likert response (0 indicating no pain, 5 indicating severe pain) giving a total score out of 20. It is a valid and reliable measure that has been used extensively in OA studies [45].

##### Global rating of change

Participants will rate their perceived overall change in their knee and their change in pain and in physical function with treatment compared to baseline on seven-point ordinal scales (1-much worse, to 7-much better). Scales of this kind are frequently used as an external criterion for comparison with changes in scores of other outcomes. Measuring patient-perceived improvement using a rating of change scale has been shown to be a clinically relevant and stable concept for interpreting truly meaningful improvements from the individual perspective [46].

##### Strength

Maximal normalized isometric quadriceps and hamstring muscle strength (peak torque; Nm/kg) at 90 degrees of knee flexion will be assessed in sitting using custom-designed apparatus comprising a force transducer (Sparker Instruments, Wenzhou, China) attached to a chair. Lever arm length will be measured as the distance from the knee joint line to the mid-point on the ankle cuff. After two submaximal warm-up trials, a total of three maximal three second trials will be performed separated by a 30 second rest period. The peak value will be used for analysis. Maximal normalized isometric hip abduction strength (peak torque, Nm/kg) will be measured in supine using a handheld dynamometer (Nicholas MMT, Lafayette Instruments, Lafayette, IN) [47]. After two, sub-maximal warm-up trials to familiarise participants with the testing procedure, participants will perform three maximal contraction trials each of three seconds duration, separated by 30 seconds of rest. The mean of the two trials will be used for analysis.

##### Functional performance

The 30-second sit-to-stand test provides a direct, objective measure of physical function [48]. After a practice trial, the number of times participants can rise to a full standing position from sitting and return to sitting, with arms crossed and held against the chest, in 30 seconds will be counted. A greater number indicates better performance.

Walking performance will be assessed by calculating walking velocity (m/sec) as participants walk 20 meters in their usual footwear with the instructions “walk as quickly as you can without overexerting yourself” [49]. This will be performed twice and the times averaged with higher velocity values indicating better walking performance.

Dynamic standing balance will be assessed by the step test. Participants are requested to stand on their most painful leg in front of a 15 cm high step. They are required to place their contralateral foot up and down on the step as many times as they can in 15 seconds. A practice trial involving 3–4 steps will be performed prior to the test trial. A greater number of steps indicates greater balance and function [50].

The timed up and go (TUG) test evaluates walking speed and mobility [51]. Participants are instructed to stand up from a standard height chair and walk at their normal pace around a marker 3 meters away before returning to the chair and sitting down again. A total of two trials will be performed and the best result taken as the final score. Faster times indicate greater performance.

##### Physical activity level

Habitual physical activity will be measured in two ways, one using a questionnaire and the second using a pedometer. The Physical Activity Scale for the Elderly (PASE) is a 10-item questionnaire that will be used to measure both the frequency and type of recreational and occupational physical activity undertaken by participants over the previous week. Higher scores indicate greater levels of physical activity. The PASE was developed and validated in samples of older adults ≥ 55 years of age [52].

A pedometer (HJ-005 Omron Healthcare, Japan) will be worn at the waist for seven consecutive days on three occasions (baseline, week 12 and week 52) to record the number of steps taken per day. Participants are requested to wear the pedometer full time during their waking hours. Pedometers have been found to be a simple and inexpensive means to estimate physical activity levels [53].

##### Health-related quality of life

This will be assessed using the Assessment of Quality of Life instrument version 2 (AQoL II) which has 20 questions covering six dimensions including independent living, social relationships, physical senses, coping, pain and psychological wellbeing [54]. The AQoL II is a multi-attribute instrument with strong psychometric properties. It produces a single utility index that ranges from −0.04 (worst possible health-related quality of life) to 1.00 (full health-related quality of life) [54].

##### Psychological measures

The Arthritis Impact Measurement Scale (AIMS2) is a disease-specific self-reported instrument designed to assess the health status of those with rheumatic conditions [55]. It is comprised of 12 subscales, of which three will be used in this study. Two are psychological subscales relating to levels of mood (5 questions) and tension (6 questions) over the past month while the third pertains to thoughts of overall arthritis impact (1 question). Questions are rated on a 5-point Guttman scale and total scores are summed with a range from 0 to 60 with higher scores indicating greater disability. The AIMS2 has high-internal consistency, test-retest reliability and validity and is moderately sensitive to change [55].

The Arthritis Self-Efficacy Scale is a 20-item questionnaire with three subscales that assess self-efficacy for control of pain management (5 questions), for physical function (9 questions) and for other arthritis symptoms (6 questions). Questions are rated on a numerical rating scale from “1” (very uncertain), to “10” (very certain). The mean value of each of the items in a subscale provides an overall score for each subscale. The range of scores for each of the subscales is from 1 to 10. Higher scores indicate high levels of perceived self-efficacy. Previous studies support the reliability and validity of this scale in those with arthritis [56].

The Arthritis Self-Efficacy Scale for Pain Communication is a modified version of the Arthritis Self-Efficacy Scale [56]. It is comprised of 5-items assessing the participants’ level of confidence in communicating their pain to their spouse/partner and their confidence that they will receive help, support and understanding from them [57]. Items are rated on a scale of “10” (very uncertain) to ”100” (very certain). Scores range from 10 to 100 and summary scores are determined by calculating the mean rating across all 5 items. This scale has been found to have good internal consistency (Cronbach α=0.94) [57].

The Pain Self-Efficacy Questionnaire is a 10-item scale measuring both the individuals’ expectation and confidence that they can perform a particular task. It covers a range of functional and non-functional tasks such as housework, socialising and coping with pain without medication. Questions are rated on a 7-point Likert scale ranging from “0” (not confident at all) to “6” (completely confident). The scores are summed to give a total score with a range of 0–60. Individuals with scores <20 are considered to have low pain self-efficacy whereas those with scores >40 are considered to have high pain self-efficacy [58]. This questionnaire is a valid measure with high internal consistency and test-retest reliability [59].

Pain catastrophising will be measured using the 13-item Pain Catastrophising this scale is divided into three subscales that measure tendencies to ruminate about pain (4 questions), magnify pain (3 questions), and feel helpless about pain (6 questions). Each item is rated on a 5-point scale ranging from “0” (not at all) to “4” (all the time). The total score is a sum of all items from each subscale with higher scores indicating greater levels of catastrophising. The scale has high internal consistency and is associated with heightened pain, psychological distress, and physical disability [60].

The Coping Strategies Questionnaire (CSQ) [61] will be administered to assess both cognitive and behavioural pain coping strategies. This 48-item self-reported questionnaire comprises a cognitive pain coping strategies component with 44 items covering 6 subscales: diverting attention, catastrophising, reinterpreting pain sensations, ignoring sensations, coping self-statements and praying and hoping. The behavioural pain coping strategies component assesses the effectiveness of the strategies above to control and decrease pain. It is comprised of 4 items containing 2 subscales: increasing pain behaviour and increasing behavioural activities. Each of the 48 items is scored on a 7-point Likert scale from “0” (never do that) to “6” (always do that). Participants also rate their overall effectiveness of the coping strategies used, how much control they feel they have over their pain and how much they feel they are able to reduce their pain. Ratings are made on a 7-point Likert scale ranging from “0” (no control/can decrease pain somewhat) to “6” (complete control/can decrease it completely) [61]. Total scores are obtained by the sum of each of the values from questions pertaining to a particular subscale for each of the cognitive or behavioural components. Higher score values indicate greater pain coping abilities. The CSQ is a commonly used instrument in both clinical and research settings [62]. It has demonstrated sensitivity to change from treatment in chronic pain samples as well as good construct validity as well as good internal consistency [61,62].

The Depression, Anxiety and Stress Subscale (DASS) measures three related negative emotional states of depression, anxiety and stress [63]. The 21-item subscale will be used instead of the full version as it is more practical for research purposes. This version has 7 questions for each of the three subscales taken directly from the DASS questionnaire. Questions consist of statements pertaining to the past week and are rated on a 4-point scale ranging from “0” (did not apply to me) to “3” (applied to me very much, or most of the time). Scores from each subscale are summed and multiplied by two. Thus, subscale scores range from 0–42 with higher scores indicating greater levels of distress. It has high internal consistency and construct validity [63,64].

The Holding Back Scale (HBS) is a modified version of the Emotional Self-Disclosure Scale (ESDS) developed by Snell et al. [65]. The HBS assesses the extent to which participants discuss their OA disease-related thoughts and feelings with their spouse/partner [66]. High levels of holding back have been found to be significantly associated with increased psychological distress and poor relationship functioning in cancer patients [66-68]. It is comprised of 11-items relating to OA-related fear and concerns, pain, body appearance, financial and job-related concerns. Those without a spouse or significant other will be advised to think of someone they are close to such as a child or friend when completing the questionnaire. Questions were rated on a 6-point Likert scale from “0” (not at all) to “5” (a lot). The sum of scores range from 0–55 for a total score with higher scores representing a greater willingness to discuss relevant emotions with a spouse or significant other [65]. The HBS is a valid and reliable measure which has high internal consistency in cancer patients [69].

The Patient Health Questionnaire-9 (PHQ9) is a short version of the Patient Health Questionnaire (PHQ) that screens for psychological disorders. The PHQ-9 is a 9-item depression scale that scores each of the 9 DSM-IV criteria. It can both establish the depressive disorder as well as determine symptom severity. Questions are rated on a 4-point scale ranging from “0” (not at all) to “3” (nearly every day). The sum of scores with a range of 0–27 determines the severity measure. Scores ≤ 15 represent none to moderate severity of depression and those ≥ 15 represent moderately severe to severe depression. It is commonly used in clinical settings and is a reliable and valid measure of depression severity [70].

Three constructs for physical activity beliefs will be assessed at baseline by reliable questionnaires. The Self-Efficacy for Physical Activity Scale is a 5-item scale that evaluates confidence in ability to participate regularly in physical activities during a variety of situations and feelings with higher scores indicating greater self-efficacy for physical activity [71]. The Benefits of Physical Activity Scale is a 14-item scale that examines whether participants are aware of the benefits of physical activity in terms of physical, psychological and social constructs with higher scores indicating a perception of more benefits [71]. The Barriers to Physical Activity Scale is a 23-item self-report questionnaire that identifies the extent to which specific conditions are considered to make participation in physical activities difficult [71]. Higher scores indicate a perception of more barriers to physical activity and have been correlated to less exercise participation [71]. In addition, a customized questionnaire relating to barriers and enablers to the home strengthening exercise program will be administered to the exercise groups.

#### *Other measures*

Participants will rate their thoughts about the treatment credibility and their treatment expectations after the first and last treatment sessions (Weeks 1 and 12) using a 4-item scale that has been previously described [72]. The first three questions are rated on a 11-point numerical rating scale from “0” (not at all confident) to “10” (absolutely confident) and pertain to the participants’ confidence in the treatment to manage and relieve their pain as well as their confidence in recommending the treatment to a friend in a similar condition. The last question assesses how logical the participant thought the treatment was and is rated on a 7-point numerical rating scale ranging from “0” (not logical at all) to “6” (very logical).

Participant adherence to the treatment program in all three groups will be obtained by recording the number of physiotherapy sessions attended (out of a maximum number of ten). Participants will maintain a daily logbook to record the number of home exercises and/or pain coping skills practice completed during the 12-week treatment phase. The physiotherapist will also indicate on an 11 point numeric rating scale, their perceived rating of the participant’s overall adherence to the treatment program with “0” (not at all) to "10" (completely as instructed) [73].

Home exercise adherence during the follow up period will be measured in the exercise only and the integrated exercise and PCST groups via a self-report questionnaire. This will be administered on four occasions (22, 32, 42 and 52 weeks). Participants will be asked how many times in the previous week they performed the home exercises. These will be summed over the four occasions (maximum of 12 exercise days). To assess the amount of home pain coping skills practice performed during the follow-up period, participants in the PCST alone group or the integrated group will be mailed a short questionnaire at weeks 22, 32, 42 and 52 which asks how many times they have performed each of the different skills practice in the previous week. Participants in all groups will provide a self-rating of their adherence to their specific home program using a similar 11 point numeric rating scale with “0” (not at all) to "10" (completely as instructed) at weeks 22, 32, 42 and 52. Furthermore, the usefulness of the various pain coping skills will be determined at weeks 32 and 52 in the PCST groups by a 5-point likert scale from “1” (not useful) to “5” (very useful).

Adverse events and the use of co-intervention will be recorded in the logbook during the treatment period and via questionnaire at weeks 12, 32, and 52 in the follow-up phase. Adverse events will be defined as any problem from the exercises and/or pain coping skills that lasted for more than two days and caused them to seek treatment.

Information on health care costs and direct non-health care costs over the entire study period (52 weeks) will be collected by questionnaire. Direct health care costs will include costs of physiotherapy attendance, additional health provider visits (doctors, specialists, other health care professionals), investigative procedures, purchase of prescription and over the counter medication, home care and hospitalization. Direct non-health care resources will include number of lost days from work.

### Sample size

The two primary endpoints are knee pain measured on the VAS and WOMAC physical function score. The minimum clinically important difference to be detected in OA trials is a change in pain of 18 mm (on 100 mm VAS) [44] and a change of six physical function WOMAC units (out of 68) [73]. Based on our previous data, we assume a common between-participant standard deviation of change of 30 mm for pain and 12 units for WOMAC physical function. These statistics indicate a smaller standardized effect size of interest (Cohen’s *d*) of 0.5 for the WOMAC measure than the *d* of 0.6 for pain. Since primary analyses will adjust for baseline of the outcome measure by ANCOVA, the sample size to detect the above effect sizes with 80% power taking into account a pre-post correlation of 0.50 is 48 patients per arm for WOMAC function and 33 per arm for VAS pain. We obtained the pre-post correlation from data from Lim et al., in which correlations of 0.58 and 0.77 were observed for pain and function respectively [74], and therefore our value of 0.50 is expected to be conservative. A further issue is that comparisons of the exercise alone arm with each of the PCST arms involve separate physiotherapists per arm and therefore clustering effects by physiotherapists need to be accounted for in the sample size and analysis. Prior data indicate that the intra-physiotherapist correlation for pain and function is likely to be at most 0.050 [74]. Assuming physiotherapists will each treat on average 7 patients (hence clustering design effect = 1 + 6*0.050 = 1.30), a total sample size of 63 patients per arm is required. Assuming 10% dropout increases the sample size to 70 per arm, or 10 physiotherapists treating 7 patients each. Slight loss of power is expected due to imbalances in number of patients per physiotherapist; however with access to 11 physiotherapists in the PCST arms this should be negated. The comparison of Exercise and PCST versus PCST alone arms will be performed ‘within-physiotherapist’ and hence does not suffer from these clustering effects. With 70 patients per arm the power is 93% to detect the WOMAC effect size and 98% for pain.

### Statistical analysis

Comparisons will be performed using an intention-to-treat analysis using all randomized participants. This analysis will include all participants including those who have missing data and those who are not fully adherent to the protocol. Some attrition is anticipated despite the fact that we will implement procedures to minimize loss to follow-up and participant withdrawal, and maximize adherence. We will employ multiple imputation methods to handle missing data in the analyses. Standard diagnostic plots will check model assumptions. Effect sizes will be calculated with an effect size of 0.2 being small, 0.5 medium and 0.8 large. All tests will be two-tailed and carried out at the 5% significance level.

Demographic and clinical characteristics as well as baseline data will be presented to assess the baseline comparability of the intervention groups. These variables will also be examined for those participants who withdraw from the study. Descriptive statistics will be presented for each group as mean change (standard deviation, 95% confidence intervals) in the two primary outcomes from baseline to 12 weeks. Between-group mean differences and 95% confidence intervals will be estimated with a mixed effects linear regression model in which physiotherapists are treated as random effects and baseline scores of the primary outcome are entered as the covariate [75], together with adjustment for the stratification variables of site and gender. Secondary outcomes will be as above for normally distributed measures, or will use binary or proportional odds random effects regression models for binary or ordinal outcomes as appropriate. Standard diagnostic plots will check model assumptions. All tests will be two-tailed and carried out at the 5% significance level with no statistical adjustment for multiple testing.

### Economic evaluation

The economic evaluation will assess the incremental cost of the integrated intervention compared with PCST and with exercise. In each case the incremental cost will be compared to the incremental benefits of treatment in terms of a clinically significant improvement in pain, a clinically significant improvement in function, and the difference in quality adjusted life years (QALYs). The incremental QALYs will be measured by the between group difference in the mean AQoL score over 12 months. A social perspective on costs will be taken and will include resource use incurred both by health services and by the participant irrespective of payment source. Health care costs will be calculated from the utilisation data and average unit costs for each item. We will not include the costs of training the physiotherapists in the delivery of PCST in the primary analysis. The inclusion of time/productivity gains is controversial and the cost effectiveness ratios will be calculated with and without these “indirect costs”. Confidence intervals for incremental cost effectiveness will be calculated directly using non parametric bootstrapping. In addition we will calculate a cost effectiveness acceptability curve based for a range of hypothetical money values of outcomes [76]. This will be done using individual cost and outcome data over the 12 months or, if adjustments for imbalance at baseline and clustering of patients by physiotherapist are necessary, analysed using a mixed linear regression model [77].

### Timelines

This study has been funded by Australian Health Management and ethics approval was obtained at the University of Melbourne in March 2010 and at the University of Queensland in June 2010. Radiation Safety approval to conduct weight bearing knee joint x-rays was approved at the Universities of Melbourne and Queensland in June 2010. Recruitment and training of the physiotherapists occurred after this time period. The anticipated timelines for the project at both sites are as follows:

August 2010 Baseline testing commences

March 2012 Recruitment complete

June 2012 All participants complete immediate post intervention testing (Week 12)

March 2013 All participants complete 9 month follow-up (Week 52)

## Discussion

Osteoarthritis is a complex chronic disease with many factors that contribute to the pain and disability. This reinforces the need for a biopsychosocial approach when developing treatments aimed at improving the self-management of this disease. Previous studies of muscle strengthening exercises have shown significant improvements in pain, physical function and performance measures [5,25,78]. However, individuals with knee OA also require treatment that addresses the psychological impairments that are commonly found [79]. An individuals’ coping mechanism for how they deal with pain is of crucial importance [80]. For instance, those who develop maladaptive coping behaviours such as limiting activity due to fear of increased joint damage and/or increased pain, avoiding social obligations, pain catastrophising and worry can lead to reports of increased pain and functional decline [80-83]. Depression, anxiety and stress are also significant predictors of upcoming pain symptoms [3,31,84]. Overwhelmingly, the research suggests that pain catastrophising and self-efficacy cognitive variables are important predictors of pain and function [13,29,85]. Keefe et al., provide support for the use of PCST in improving psychological functioning in this patient population [24,34,35,81]. Thus, given that both exercise and PCST have been shown to be effective for symptomatic relief in knee OA, we contend that an integrated PCST and exercise program is likely to be more efficacious than either intervention alone.

The study design has several major strengths. First, considerable attention has been paid to quality control of the PCST intervention. The project physiotherapists will undergo rigorous training and accreditation to deliver the PCST intervention. All sessions will be audio recorded and then reviewed by the site psychologist. Regular meetings will be held throughout the study to provide ongoing practice and supervision for the physiotherapists. PCST treatment fidelity will be formally determined via rating of a random sample of the PCST treatments. Second, the outcome measures used are valid and reliable, cover a range of clinically important constructs and include those recommended for clinical trials of OA [44]. These include self-report measures of pain, function, quality of life and global response to treatment. A range of other measures is also included to encompass functional performance, strength, psychological functioning and physical activity levels. In addition, a health economic assessment is included given the need to justify the cost-effectiveness of treatments in the current economic climate. Third, the study has been designed with attention to methodological features such as randomization, concealed allocation and blinded outcome assessment. The sample size has been calculated to detect minimal clinically important differences between groups, taking into account the clustering effects of the physiotherapists.

## Conclusion

This study uses a randomized controlled trial design to investigate the efficacy of an integrated exercise and PCST program delivered by physiotherapists in improving pain and physical function in those with knee OA compared with exercise or PCST alone. The novel findings will enable evidence-based recommendations as to the efficacy of this conservative option for the management of patients with knee OA.

## Competing interests

The authors declare that they have no competing interests.

## Authors’ contributions

KLB, FJK conceived the project and KLB is leading the co-ordination of the trial. KLB, FJK, CB, GJ, MH, JK, MN, AF and AH designed the protocol and procured the project funding. FJK developed the pain coping skills training protocol, developed and ran the 4-day workshop to train the physiotherapists and consults with the site psychologists regarding the protocol on an as needed basis. GJ is leading the Brisbane study site. GJ, KLB and MH designed the exercise intervention and GJ and KLB were responsible for training the physiotherapists in the exercise protocol and grading x-rays for participant inclusion. YA is a PhD student responsible for managing the Melbourne study site and for recruiting and screening participants. CB and JK are the study psychologists involved in overseeing the site psychologists. AF provided the sample size calculations, designed the statistical analysis and provided the randomization schedule. AH designed the economic evaluation. TE and BM assisted with development of study material and are blinded assessors. BM set up the initial study database. All authors provided feedback on drafts of this paper and read and approved the final manuscript.

## Pre-publication history

The pre-publication history for this paper can be accessed here:

http://www.biomedcentral.com/1471-2474/13/129/prepub
